# University of Louisville

**Published:** 1852-07

**Authors:** 


### UNIVERSITY OF LOUISVILLE.

Professor Drake's devotion to Cincinnati, and to the school of his earlier days, has again prevailed with him to resign his chair in the University of Louisville, and return to the Medical College of Ohio, and Professor Cobb has followed his example. The paramount consideration with the latter gentleman for giving up a place which he had so long and so creditably held, was the opportunity afforded by the change of securing an appointment—that of Demonstrator of Anatomy—for his promising son, Dr. William H. Cobb. The separation of those gentlemen from their old associates is without any diminution of that mutual esteem and good will, which has subsisted between them during all their professional intercourse.

The Board of Trustees have filled the vacancies by the appointment of Professor Austin Flint, of the University of Buffalo, to the chair of *Theory and Practice*, and of Professor Benjamin R. Palmer, to the chair of *Anatomy*. Professor Palmer is connected with the University of Buffalo, as Professor of Anatomy, and has for a number of years held the same chair in the Medical College of Vermont, at Woodstock.

We have delayed the issue of our present number a few days for the purpose of being able to make this announcement, and have now space for little more than to congratulate the alumni and friends of the University upon the very satisfactory manner in which the Board have supplied the places of Profs. Drake and Cobb. Like their predecessors, Professor Flint and Professor Palmer are men of a national reputation, well tried and eminently successful as teachers. As a lecturer, those who have been associated with him speak of Professor Palmer in the highest terms. His lectures and demonstrations are described as being clear, accurate, thorough, and his manner one to awaken enthusiasm in the mind of the student, secure his unremitted attention, and enforce the acquisition of anatomical knowledge. Professor Flint at one time held a chair in the Medical College of Chicago, and has been for a number of years Professor of the Theory and Practice of Medicine in the University of Buffalo. His experience both in private and hospital practice has been ample, and he is known everywhere in our country as a writer on practical medicine. His essay "*On Variations of Pitch in Percussion and Respiratory Sounds*," as has already been noticed in this journal, received the prize at the late meeting of the American Medical Association, and was declared by the committee to have enlarged the bounds of medical science. He is a polished, vigorous, and original writer, and a clear, instructive, and eloquent lecturer. The accession of such men to the school is at once the best evidence of its high character abroad, and the surest guaranty of its future success.

These gentlemen have accepted.

---

UNIVERSITY OF NASHVILLE.

